# The Presence of Infective Material in Dwellings Occupied by Consumptive Persons

**Published:** 1901-07-27

**Authors:** 


### The Presence of Infective Material in Dwellings Occupied by Consumptive Persons.

Dr. Harold Coates, senior assistant medical officer of health for the City of Manchester, addressing the Congress on Wednesday on the results of the investigations which he had conducted, held that living and virulent tubercle bacilli are present in the dust which has deposited itself naturally from the atmosphere in rooms which are occupied by phthisical patients who are in the habit of spitting upon the floor or into pocket-handkerchiefs, and that this infective dust is present in greatest amount or is most virulent where the access of sunlight and the free circulation of air are prevented. Such infected rooms must be a source of danger to those occupying them. Healthy persons run the risk of being infected, and the patient himself may become reinfected and his ultimate recovery be delayed or prevented. It is well known that the tubercle bacillus can retain its virulence for months in the dry state; it is evident, therefore, that these houses will remain infective for some considerable time after a patient leaves it. Unless there are some measures taken to remove this infective dust, a new tenant coming into a house lately occupied by a consumptive patient is exposed to the danger of acquiring phthisis. In the poorer quarters of large cities, where houses are too few for the needs of the population, the new tenant often takes possession the same or the following day that the late tenant leaves, and nothing in the way of disinfection of cleansing the walls and ceilings is done. There is no doubt that many persons are in this way unknowingly exposed to infection, and that an infected dwelling is in many cases the origin of a case of phthisis which the most careful inquiry has failed to trace to any direct association with a previous case. The same danger exists to some extent at the boarding houses and hotels at health resorts.

